# Hepatitis B Virus X Protein Induces Reactive Oxygen Species Generation via Activation of p53 in Human Hepatoma Cells

**DOI:** 10.3390/biom14101201

**Published:** 2024-09-24

**Authors:** Seungyeon Kim, Jimin Park, Jiwoo Han, Kyung Lib Jang

**Affiliations:** 1 Department of Integrated Biological Science, The Graduate School, Pusan National University, Busan 46241, Republic of Korea; hatmddus135@korea.kr (S.K.); gi609gi@pusan.ac.kr (J.P.); hanjiwoo@pusan.ac.kr (J.H.); 2 Department of Microbiology, College of Natural Science, Pusan National University, Busan 46241, Republic of Korea; 3 Microbiological Resource Research Institute, Pusan National University, Busan 46241, Republic of Korea

**Keywords:** HBx, hepatitis B virus, proteasome, reactive oxygen species, p53

## Abstract

Hepatitis B virus (HBV), particularly through the HBx protein, induces oxidative stress during liver infections. This study reveals that HBx increases reactive oxygen species (ROS) via two distinct mechanisms. The first mechanism is p53-independent, likely involving mitochondrial dysfunction, as demonstrated by elevated ROS levels in p53-deficient Hep3B cells and p53-knocked-down HepG2 cells after HBx expression or HBV infection. The increase in ROS persisted even when p53 transcriptional activity was inhibited by pifithrin-α (PFT-α), a p53 inhibitor. The second mechanism is p53-dependent, wherein HBx activates p53, which then amplifies ROS production through a feedback loop involving ROS and p53. The ability of HBx to elevate ROS levels was higher in HepG2 than in Hep3B cells. Knocking down p53 in HepG2 cells lowered ROS levels, while ectopic p53 expression in Hep3B cells raised ROS. HBx-activated p53 downregulated catalase and upregulated manganese-dependent superoxide dismutase, contributing to ROS amplification. The transcriptional activity of p53 was crucial for these effects, as cells with a p53 R175H mutation or those treated with PFT-α generated less ROS. Additionally, HBx variants with Ser-101 increased p53 and ROS levels, whereas variants with Pro-101 did not. These dual mechanisms of HBx-induced ROS generation are likely significant in the pathogenesis of HBV and may contribute to liver diseases, including hepatocellular carcinoma.

## 1. Introduction

Hepatitis B virus (HBV) is a significant human pathogen, responsible for approximately 300 million chronic liver disease cases worldwide [1,2]. As a member of the *Hepadnaviridae* family, HBV replicates and encapsidates a partially double-stranded circular DNA genome of approximately 3200 base pairs through reverse transcription of a pre-genomic RNA [3]. Of the four open reading frames (S, C, P, and X) in the HBV genome, the shortest is X, which encodes the 17-kDa HBV X protein (HBx). HBx is a multifunctional oncoprotein found in the cytoplasm, nucleus, and mitochondria, influencing several cellular processes such as signal transduction, transcription, and mitochondrial function [1,2]. Additionally, HBx acts as a positive regulator of HBV replication by activating the four viral promoters, thereby enhancing the synthesis of HBV mRNA and pre-genomic RNA [3]. Therefore, HBx is essential for both HBV replication and viral pathogenesis.

Reactive oxygen species (ROS) levels are typically elevated in the liver and blood of patients infected with HBV [4,5,6]. Both host and viral factors contribute to this phenomenon. Virus-specific cytotoxic T lymphocytes play a major role in ROS generation by eliminating infected hepatocytes and stimulating inflammatory cytokines [7]. Additionally, HBV itself induces ROS production during infection, primarily through the HBx protein [8,9,10]. HBx interacts with a voltage-dependent anion channel in the mitochondria, altering its transmembrane potential, and downregulates mitochondrial enzymes involved in electron transport, leading to increased mitochondrial ROS levels [11,12,13,14,15]. However, the detailed mechanism of HBx-induced ROS generation remains unclear.

Antioxidant enzymes are involved in the catalytic transformation of ROS and their by-products into stable nontoxic molecules [16]. The main intracellular antioxidant enzymes include superoxide dismutase (SOD), catalase, and glutathione peroxidase (GPx), which together regulate ROS levels in mammalian cells [17]. In the antioxidant enzyme system, SOD catalyzes the conversion of superoxide radicals to hydrogen peroxide (H_2_O_2_), which is further metabolized to water (H_2_O) and oxygen (O_2_) by catalase and GPx. An imbalance in the coordinated expression or activity of these enzymes can lead to the generation of oxidative stress [18]. For instance, even minor deviations in the GPx/SOD activity ratio can significantly affect cellular resistance to oxidative damage [18].

The tumor suppressor protein p53 is a redox-active transcription factor that orchestrates cellular responses against various stresses causing genomic instability [19]. In response to DNA damage caused by excessive ROS, p53 can regulate ROS levels through a positive feedback loop by activating specific target genes [20,21,22]. Notably, p53 controls the expression of antioxidant enzymes such as GPx, manganese-dependent SOD (Mn-SOD), and catalase [23,24,25]. Given that HBx increases p53 levels through the ATM-Chk2 pathway [26], this study investigates whether HBx-induced ROS affects antioxidant enzyme expression via the activation of p53, thus amplifying ROS through a feedback loop. Utilizing an optimized in vitro HBV replication system [27], the study first examines if HBV-induced ROS generation varies based on p53 status in human hepatoma cells. It then explores how HBx induces ROS amplification in a p53-dependent manner. The study further investigates whether the transcriptional activity of p53 is crucial for HBx-mediated ROS amplification and examines if HBx regulates catalase, Mn-SOD, and GPx expression in a p53-dependent manner to enhance ROS levels during both HBx expression and HBV infection.

## 2. Materials and Methods

### 2.1. Plasmids

The plasmid pCMV-3 × HA1-HBX3 (HBX3 or HBx) and pCMV-3 × HA1-hbx2 (hbx2) encode full-length HBx (genotype D) downstream of the three copies of the influenza virus haemagglutinin (HA) [28]. For the construction of the hbx2 P101S expression plasmid, Pro-101 of hbx2 in pCMV-3 × HA1-hbx2 was substituted with Ser-101 using PCR-directed mutagenesis. The 1.2-mer wild type (WT) HBV replicon containing 1.2 units of the HBV genome (genotype D) and its HBx-null counterpart [29] were kindly provided by W. S. Ryu (Yonsei University, Seoul, Republic of Korea). The plasmid RC210241, encoding the human NTCP, was obtained from OriGene (Rockville, MD, Cat No. 003049). Scrambled (SC) short hairpin RNA (shRNA) (Cat No. sc-37007) and p53 shRNA (Cat No. sc-29435) were purchased from Santa Cruz Biotechnology (Santa Cruz, CA, USA). The pCMV p53-WT, encoding WT p53, and pTG13, containing 13 copies of the p53 binding site were gifts from Chang-Woo Lee (Sungkyunkwan University, Suwon, Republic of Korea). pCMV p53 R175H plasmid was kindly provided by Bum-Joon Park (Pusan National University, Busan, Republic of Korea). The pHA-Ub encoding HA-tagged Ub was kindly provided by Y. Xiong (University of North Carolina at Chapel Hill, NC, USA). Plasmid pCH110 (Cat No. 27-4508-01), encoding the *Escherichia coli* β-galactosidase (β-Gal) gene, was obtained from Addgene (Watertown, MA, USA).

### 2.2. Cell Culture and Transfection

The cell lines HepG2 (Cat No. 88065) and Hep3B (Cat No. 88064) were obtained from the Korean Cell Line Bank (KCLB, Seoul, Republic of Korea). Stable cell lines, HepG2-NTCP and Hep3B-NTCP, were established by transfection with RC210241, followed by selection with 500 μg/mL G418 sulfate (Sigma-Aldrich, St. Louis, MO, USA, Cat No. A1720). Cells were cultured in Dulbecco Modified Eagle Medium (DMEM) (WelGENE, Gyeongsan, Republic of Korea, Cat No. LM001-05) supplemented with 10% fetal bovine serum (FBS, Capricorn Scientific, Ebsdorfergrund, Germany, Cat No. FBS-22A), 100 units/mL penicillin G (Sigma-Aldrich, Cat No. P3032), and 100 μg/mL streptomycin (United States Biological, Salem, MA, USA, Cat No. 21865) at 37 °C in 5% CO_2_-humidified atmosphere. For transient expression, 2 × 10^5^ cells per well in a 6-well plate were transfected using TurboFect transfection reagent (Thermo Fisher Scientific, Waltham, MA, USA, Cat No. R0532) according to the manufacturer’s instructions with the designated amounts of plasmids and an empty vector supplemented to make the final amount of cocktails equivalent. The cells were treated with N-acetyl-L-cysteine (NAC) (Sigma-Aldrich, Cat No. A7250), myrcludex-B (MedchemExpress, Monmouth Junction, NJ, USA, Cat No. HY-P3465), or pifithrin-α (PFT-α) (Sigma-Aldrich, P4359) under the indicated conditions.

### 2.3. Luciferase Assay

Approximately 1 × 10^5^ cells per well in 12-well plates were transfected with 0.3 μg of pHBV-luc with the indicated plasmids under the indicated conditions. To control for transfection efficiency, 0.1 μg pCH110 was co-transfected as an internal control. At 48 h after transfection, a luciferase assay was performed using the Luciferase Reporter 1000 Assay System (Promega, Madison, WI, USA, Cat No. E4550). Luciferase activity was measured using a microplate luminometer (LuBi, MicroDigital, Seongnam, Republic of Korea). β-gal activity was measured using a β-gal assay kit (Thermo Fisher Scientific, Cat No. 34055). Luciferase activity was normalized to the β-gal activity measured in the corresponding cell extracts.

### 2.4. HBV Cell Culture System

Hep3B-NTCP cells were transiently transfected with the 1.2-mer HBV replicon plasmid as described above. The culture supernatant was collected to prepare HBV seeds. HBV titers were determined by qPCR, as described in the next section. For the preparation of HBV stocks, Hep3B-NTCP cells were infected with HBV at a multiplicity of infection (MOI) of 10 for 24 h. Following infection, the medium was replaced with DMEM containing 4% PEG 8000 (Sigma-Aldrich, Cat No. D4463), 2% DMSO (Sigma-Aldrich, Cat No. D8418), and 3% FBS. The culture medium was collected every three days to prepare viral stocks. HBV infection was conducted in 6-well plates at an MOI of 50 for 4 days, unless otherwise indicated, according to an optimized HBV cell culture system with slight modifications [27]. Briefly, 2 × 10^5^ cells were inoculated with 1 × 10^7^ genome equivalents (GEQs) of HBV and incubated for 24 h as above. After washing twice with PBS, the cells were incubated in DMEM supplemented with 3% FBS, 4% PEG 8000, and 2% DMSO for an additional three days.

### 2.5. Quantitative Real-Time PCR of HBV DNA

The extracellular and intracellular HBV DNA was measured by quantitative real-time PCR (qPCR), as described previously [30]. Briefly, HBV genomic DNA was extracted from the culture supernatant using the QIAamp DNA Mini Kit (Qiagen, Hilden, Germany, Cat No. 51306). For immunoprecipitation-coupled PCR (IP-PCR), the culture supernatant was first immunoprecipitated using an anti-HBsAg antibody, as described previously [30]. For conventional PCR analysis, HBV genomic DNA was amplified using 2 × Taq PCR Master mix 1 (BioFACT, Daejeon, Republic of Korea, Cat No. ST301-19h) and a primer pair, HBV 1399F (5′-TGG TAC CTC CGC GGG ACG TCC TT-3′) and HBV 1632R (5′-AGC TAG CGT TCA CGG TGG TCT CC-3′). For qPCR analysis, HBV DNA was amplified using the SYBR premix Ex Taq II (Takara Bio, Kusatsu, Shiga, Japan, Cat No. RR82LR) and HBV 379F (5′-GTG TCT GCG TTT TAT CA-3′) and HBV 476R (5′-GAC AAA CGG GCA ACA TAC CTT-3′) using a Rotor-Gene qPCR machine (Qiagen).

### 2.6. HBV e Antigen Enzyme-Linked Immunosorbent Assay

For quantitative analysis of secreted HBV e antigen (HBeAg), 30 μL of culture supernatant was loaded onto 96-well plates using an enzyme-linked immunosorbent assay (ELISA) kit designed for the detection of HBeAg, following the manufacturer’s instructions (Cusabio, Houston, TX, USA, Cat No. CSB-E13557h). HBeAg levels were determined using a microplate reader by measuring the optical density of each well at 450 nm (Bio-Rad, Hercules, CA, USA).

### 2.7. Determination of Intracellular ROS Levels

Intracellular ROS levels were measured using chloromethyl dichlorodihydrofluorescein diacetate (CM-H_2_DCFDA; Invitrogen, Waltham, MA, USA, Cat No. C6827), which is widely applied as an H_2_O_2_-specific probe in intact cells [28]. Briefly, 1 × 10^5^ cells per well in 12-well plates were treated with 10 µM CM-H_2_DCFDA in serum-free media for 30 min. After washing with PBS, cells were collected by treatment with Trypsin-EDTA (Gibco, Cleveland, TN, USA, Cat No. 25200-072). The oxidation of CM-H_2_DCFDA to a green fluorescent product, DCF, was then quantified using a microplate reader (Mithras LB940, Berthold Technologies, Bad Wildbad, Germany) at excitation and emission wavelengths of 485 nm and 535 nm, respectively.

### 2.8. Western Blot Analysis

Cells were lysed in buffer (50 mM Tris-HCl, pH 8.0, 150 mM NaCl, 0.1% SDS, and 1% NP-40) supplemented with protease inhibitors (Roche, Basel, Switzerland, Cat No. 11836153001). Protein concentrations of cell extracts were measured using a protein assay kit (Bio-Rad, Cat No. 5000006). Cell extracts were separated by SDS-PAGE and proteins transferred onto a nitrocellulose blotting membrane (Amersham, UK, Cat No. 10600003) were incubated with primary antibodies against p21 (Santa Cruz Biotechnology, Cat No. sc-6246, 1:200 dilution), Catalase (Santa Cruz Biotechnology, Cat No. sc-271803, 1:500 dilution), Mn-SOD (Santa Cruz Biotechnology, Cat No. sc-137254, 1:500 dilution), p53 (Santa Cruz Biotechnology, Cat No. sc-126, 1:1000 dilution), HBV surface antigen (HBsAg) (Santa Cruz Biotechnology, Cat No. sc-53300, 1:400 dilution), γ-tubulin (Santa Cruz Biotechnology, Cat No. sc-17787, 1:500 dilution), and HA (Santa Cruz Biotechnology, Cat No. sc-7392, 1:500 dilution); and HBx (Millipore, Burlington, MA, USA, Cat No. MAB8419, 1:1000 dilution), and GPx (Santa Cruz Biotechnology, Cat No. sc-133160, 1:500 dilution), followed by their subsequent incubation with HRP-conjugated anti-mouse secondary antibody (Bio-Rad, Cat No. BR170-6516, 1:3000 dilution) and anti-rabbit IgG (H + L)-HRP (Bio-Rad, Cat No. BR170-6515, 1:3000 dilution). An ECL kit (Advansta, San Jose, CA, USA, Cat No. K-12043-D20) was used to visualize protein bands using the ChemiDoc XRS imaging system (Bio-Rad).

### 2.9. Quantification of Western Blot Images

Western blot images were captured using a standardized protocol to ensure consistent lighting and exposure across all samples. Band intensity quantification was performed using ImageJ software (version 2.1.0, National Institutes of Health, Bethesda, MD, USA). Each band of interest was outlined with a uniformly sized rectangular selection tool to measure the integrated density, which includes the area and mean gray value of the selected region. Background intensity was determined by measuring a similarly sized area adjacent to the bands. This background value was subtracted from the corresponding band intensity to correct for ambient noise and nonspecific signals. The normalized intensity of each protein band was then calculated relative to the intensity of the housekeeping protein γ-tubulin, providing a relative value of protein expression level in each sample.

### 2.10. Statistical Analysis

The values indicate mean ± standard deviation from at least three independent experiments. A two-tailed Student’s *t*-test was used for all statistical analyses. A *p*-value > 0.05 was considered statistically non-significant, whereas a *p*-value ≤ 0.05 was considered statistically significant.

## 3. Results

### 3.1. HBV Infection Induces Higher Levels of ROS Generation in the Presence of p53

**Figure 1:**
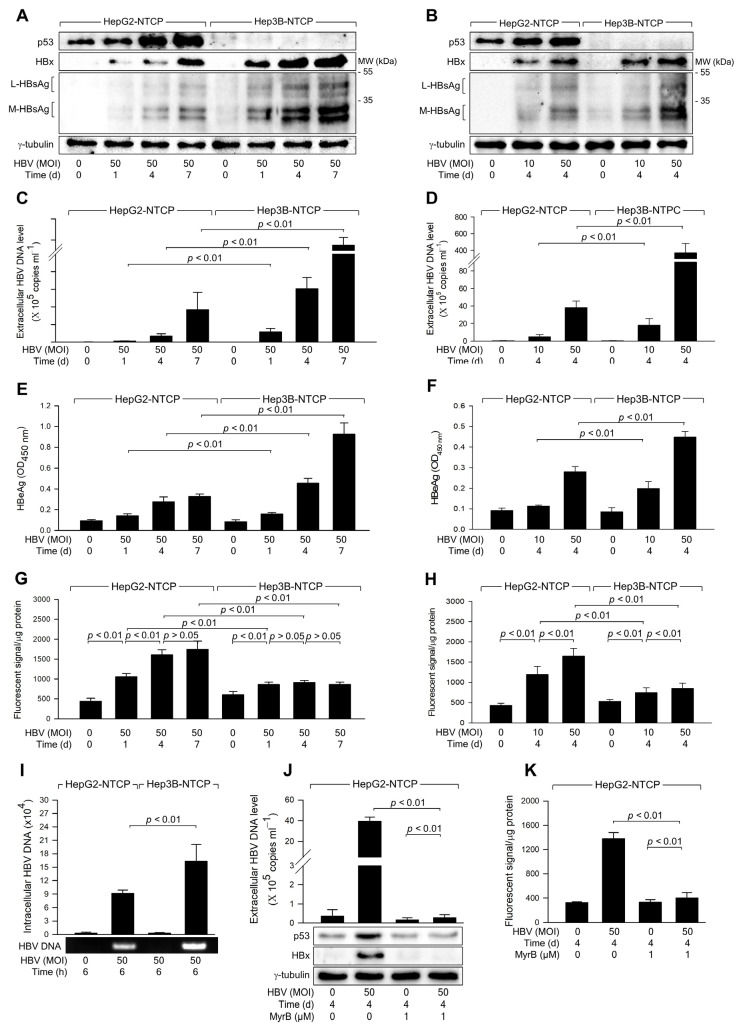
HBV infection induces higher levels of ROS generation in the presence of p53. HepG2-NTCP and Hep3B-NTCP cells were infected with HBV at the indicated MOI for 24 h in DMEM containing 2% DMSO and 4% PEG 8000, washed twice with serum-free DMEM, and then incubated for the indicated time in DMEM containing 3% FBS, 2% DMSO, and 4% PEG 8000. (**A**,**B**) Cell lysates were subjected to western blotting to measure p53, HBx, HBsAg, and γ-tubulin levels. (**C**,**D**) The levels of HBV particles released from the cells prepared in (**A**,**B**) were measured by quantitative real-time PCR (qPCR). Results are shown as mean ± standard deviation from seven independent experiments (*n* = 7). (**E**,**F**) Levels of HBeAg released from the cells prepared in (**A**,**B**) were determined by ELISA (*n* = 5 for (**E**) and 4 for (**F**)). (**G**,**H**) Levels of intracellular ROS were determined after treating cells with a fluorescent dye, CM-H_2_DCFDA (*n* = 5 for (**G**) and 10 for (**H**)). (**I**) To assess HBV internalization, cells were infected with HBV at an MOI of 50 for 6 h, following measurement of intracellular HBV DNA levels by qPCR (*n* = 3). (**J**) HepG2-NTCP cells were infected with HBV for 4 days in the presence and absence of myrcludex-B, and the levels of extracellular HBV DNA, along with intracellular protein levels, were measured (*n* = 3). (**K**) Levels of intracellular ROS were measured from the cells obtained in (**J**) as described in (**G**,**H**) (*n* = 3). Original western blots are available in the Supplementary Materials.

Two human hepatoma cell lines, HepG2 and Hep3B cells, differ primarily in their p53 status, with HepG2 expressing wild-type p53 and Hep3B lacking p53, making them valuable models for studying p53-related mechanisms in HBV research [28,31,32]. For in vitro HBV replication studies, HBV infection was conducted in HepG2-NTCP and Hep3B-NTCP cells, which stably express the HBV receptor sodium-taurocholate co-transporting polypeptide (NTCP) [33], using HBV particles derived from a 1.2-mer HBV replicon. Several methods were employed to confirm the replication of HBV replication in these cells: western blot analysis of viral proteins like HBx and HBsAg in cell lysates (Figure 1A,B), qPCR measurement of virus particles in culture supernatants (Figure 1C,D), and detection of extracellular HBeAg by ELISA (Figure 1E,F). Large (L)- and middle (M)-HBsAg were specifically detected in infected cells (Figure 1A,B), consistent with previous studies [30]. Notably, trace amounts of HBsAg from the integrated HBV genome [34] were detected in uninfected Hep3B-NTCP cell lysates (Figure 1A,B); however, no HBV replication was detected by qPCR or ELISA in these cells (Figure 1C–F). These findings confirm successful HBV replication in HepG2-NTCP and Hep3B-NTCP cells under the experimental conditions.

In line with prior studies identifying p53 as a negative regulator of HBV propagation [26], both time- and dose-dependent experiments consistently showed higher HBV replication rates in Hep3B-NTCP cells compared to HepG2-NTCP cells (Figure 1A–F). Further investigation focused on whether HBV induces ROS generation differentially depending on the p53 status of the host cells. HBV was found to increase ROS levels in both HepG2-NTCP and Hep3B-NTCP cells in a dose-dependent manner (Figure 1H). In addition, HBV induced time-dependent ROS generation in HepG2-NTCP but not in Hep3B-NTCP cells (Figure 1G). Notably, HBV consistently induced higher levels of ROS in HepG2-NTCP cells than in Hep3B-NTCP cells (Figure 1G,H). These results suggest that p53 enhances the capacity of HBV to induce ROS generation in human hepatoma cells. To investigate whether the differential replication of HBV observed in Hep3B-NTCP and HepG2-NTCP cells is due to variations in susceptibility to HBV infection, we measured HBV internalization following a short-term infection in both cell lines. After infecting cells with HBV for 6 h, the levels of intracellular viral DNA in both cell lines were quantified. The results revealed that HBV internalization was approximately twofold higher in Hep3B-NTCP cells compared to HepG2-NTCP cells (Figure 1I). This increase in viral internalization may be due to p53 expressed in HepG2 cells, which is known to downregulate levels of EGFR, responsible for the entry into cells [35,36,37]. Therefore, the higher HBV replication efficiency in Hep3B-NTCP cells is likely attributable to both HBV entry and HBx-mediated replication processes [26], both of which are negatively influenced by p53. To further assess the role of HBV replication in the modulation of p53 and ROS induction, HepG2-NTCP cells were treated with myrcludex-B, a known inhibitor of HBV entry. Following treatment, we observed a significant reduction in HBV replication, confirming the efficacy of myrcludex-B in blocking viral entry (Figure 1J). Indeed, when HBV entry was blocked, the characteristic HBV-induced increase in intracellular p53 levels was not observed (Figure 1J). Additionally, intracellular ROS levels were not elevated following myrcludex-B treatment (Figure 1K). These results indicate that HBV specifically induces p53 upregulation, as well as the subsequent increase in ROS production in HepG2-NTCP cells.

### 3.2. HBx Is Responsible for the Upregulation of ROS Levels during HBV Replication

**Figure 2:**
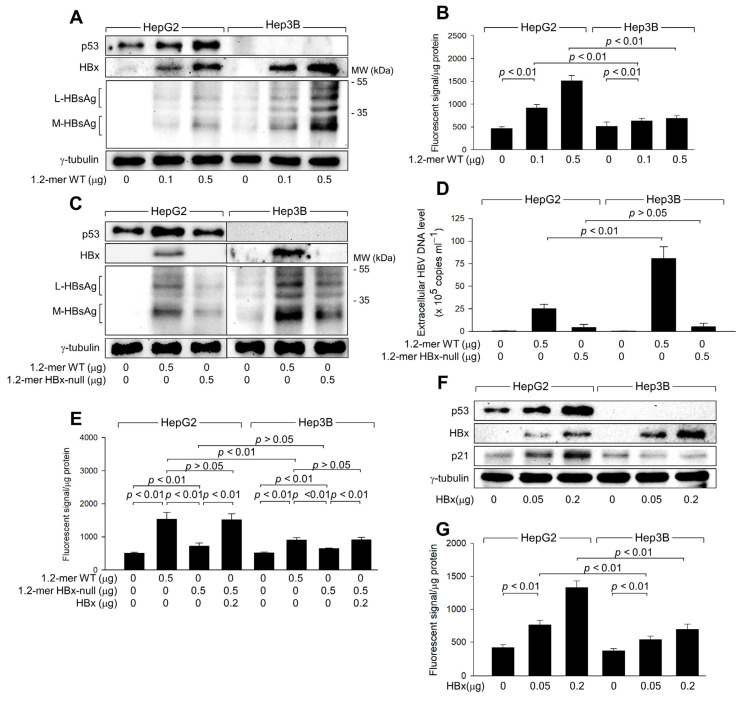
HBx is responsible for the upregulation of ROS levels during HBV replication. HepG2 and Hep3B cells were transfected with a 1.2-mer WT HBV replicon (1.2-mer WT) or its HBx-null counterpart (1.2-mer HBx-null), along with or without an HBx expression plasmid for 48 h. (**A**,**C**,**F**) Levels of the indicated proteins were determined by western blotting. (**D**) Levels of extracellular HBV DNA from (**C**) were detected with immunoprecipitation PCR using HBx antibody (*n* = 6). (**B**,**E**,**G**) Intracellular ROS levels were determined as described in Figure 1G (*n* = 7 for (**B**) and 4 for (**E**,**G**)). Original western blots are available in the Supplementary Materials.

We investigated whether HBx is responsible for the upregulation of ROS levels during HBV replication in human hepatoma cells. For this study, we used a 1.2-mer wild-type HBV replicon (1.2-mer WT) and its HBx-null counterpart (1.2-mer HBx-null) [29]. Transient transfection with 1.2-mer WT yielded higher levels of HBV proteins, including HBx and HBsAg, in Hep3B cells compared to HepG2 cells (Figure 2A). In addition, IP-PCR assays detected higher levels of HBV particles from Hep3B cells (Figure 2D). Additionally, transfection with 1.2-mer WT induced ROS generation in HepG2 cells in a dose-dependent manner, while this effect was significantly lower in Hep3B cells (Figure 2B). These results indicate that the p53-dependent ROS generation observed in an in vitro HBV infection system (Figure 1) can be precisely replicated using the 1.2-mer HBV replicon system.

Transfection with 1.2-mer HBx-null resulted in lower levels of intracellular HBsAg and extracellular HBV DNA particles in both HepG2 and Hep3B cells compared to 1.2-mer WT (Figure 2C,D), highlighting the essential role of HBx in HBV replication [3,26]. In addition, the potential of 1.2-mer HBx-null to induce ROS generation was significantly reduced in both cell lines compared to 1.2-mer WT, with a more pronounced difference in HepG2 cells (Figure 2E). The defect in ROS generation by 1.2-mer HBx-null was almost entirely restored by ectopic expression of HBx in both HepG2 and Hep3B cells (Figure 2E), indicating that HBx is involved in both p53-dependent and independent ROS generation during HBV infection. To confirm the direct role of HBx in ROS generation, we transiently transfected HBx into HepG2 and Hep3B cells (Figure 2F). Ectopic expression of HBx alone was sufficient to induce ROS generation in both HepG2 and Hep3B cells in a dose-dependent manner (Figure 2G). Although HBx significantly increased ROS levels in Hep3B cells, the effect was less pronounced compared to HepG2 cells (Figure 2G). These observations confirm that HBx plays a central role in both p53-dependent and p53-independent ROS generation during HBV infection in human hepatoma cells.

### 3.3. HBx Is Responsible for the Upregulation of ROS Levels during HBV Replication

We investigated how HBV induces ROS generation in human hepatoma cells. Consistent with previous reports [26], HBV infection upregulated p53 levels in HepG2-NTCP cells in a time- and dose-dependent manner (Figure 1A,B). The 1.2-mer WT HBV replicon, but not the 1.2-mer HBx-null variant, similarly upregulated p53 levels in HepG2 cells (Figure 2A,C). Additionally, ectopic expression of HBx increased p53 levels, leading to the upregulation of p21, a representative p53 target, in these cells (Figure 2E). Moreover, the strong correlation between p53 and ROS levels observed under various conditions (Figure 1 and Figure 2) suggests a critical role for p53 in ROS generation during HBV replication.

**Figure 3:**
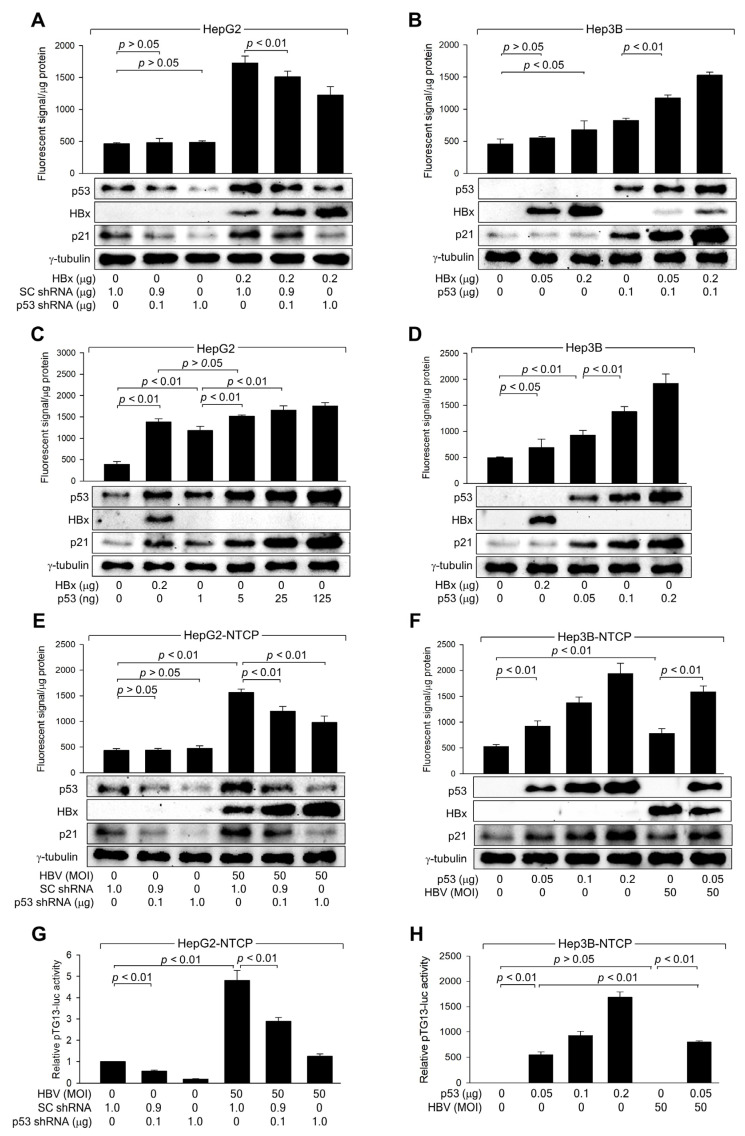
HBx increases ROS levels by activating p53 during HBV replication. (**A**–**D**) HepG2 and Hep3B cells were transfected with the indicated amounts of HBx expression plasmid along with scrambled (SC) shRNA, p53 shRNA, or p53 expression plasmid for 48 h, followed by western blotting and measurement of intracellular ROS levels (*n* = 4 for (**A**–**C**) and 5 for (**D**)). (**E**,**F**) HepG2-NTCP and Hep3B-NTCP cells were transfected with the indicated plasmid for 24 h, followed by infection with HBV for an additional 24 h. Afterward, the infection medium was replaced, and the cells were incubated for an additional 3 days. Following this incubation, cells were harvested for western blot analysis and intracellular ROS level measurement (*n* = 4). (**G**,**H**) HepG2-NTCP and Hep3B-NTCP cells were transfected with pTG13-luc, which contains 13 copies of the p53 binding site, along with the indicated plasmids. Following infection with HBV for 24 h, the medium was replaced, and the cells were incubated for an additional 3 days. Afterward, a luciferase assay was performed, and the results are presented as relative luciferase activity compared to the basal level of the control (*n* = 4). Original western blots are available in the Supplementary Materials.

To further explore the role of p53 in ROS generation, p53 was knocked down in HepG2 cells using p53 shRNA, whereas ectopic p53 expression was used to overexpress p53 in Hep3B cells. p53 knockdown in HepG2 cells, both with and without HBx, led to decreased p53 and p21 levels, while HBx levels increased in a dose-dependent manner (Figure 3A). This reduction in p53 levels corresponded with lower ROS levels in the presence of HBx, whereas the effect was minimal without HBx, likely due to the limits of ROS detection (Figure 3A). Although HBx could induce ROS generation independently of p53, the effect was significantly enhanced in the presence of ectopic p53, which was also upregulated by HBx (Figure 3B).

Next, it was investigated whether HBx-induced activation of p53 is sufficient to induce ROS generation in human hepatoma cells. Ectopic expression of p53 alone, without HBx, could induce ROS generation in both HepG2 and Hep3B cells in a dose-dependent manner (Figure 3C,D). Notably, comparable levels of p21 and ROS were observed when similar levels of p53 were generated in HepG2 cells by transfection with either an HBx expression plasmid or a p53 expression plasmid (Figure 3C). Based on these observations, we conclude that HBx induces intracellular ROS generation by elevating p53 levels in human hepatoma cells.

Consistent with data obtained with HBx overexpression (Figure 3A,B), p53 knockdown specifically reduced p53 expression and activity in HepG2-NTCP cells, both with and without HBV infection, resulting in lower p21 levels (Figure 3E,G). This knockdown strongly inhibited ROS generation in HBV-infected HepG2-NTCP cells, whereas the effect was negligible in mock-infected cells (Figure 3E). Moreover, while either ectopic p53 expression or HBV infection individually increased ROS levels in Hep3B-NTCP cells, their combination led to a more dramatic increase simply because HBx upregulated the protein level and transcription activity of ectopic p53 (Figure 3F,H). These findings confirm that p53 amplifies ROS levels during HBV infection. Additionally, p53 knockdown increased HBx levels in HBV-infected HepG2-NTCP cells, whereas p53 expression decreased HBx levels in HBV-infected Hep3B-NTCP cells (Figure 3E,F), confirming the role of p53 as a negative regulator of HBV replication in human hepatoma cells.

### 3.4. HBx Requires p53 Transcriptional Activity to Amplify ROS Levels

**Figure 4:**
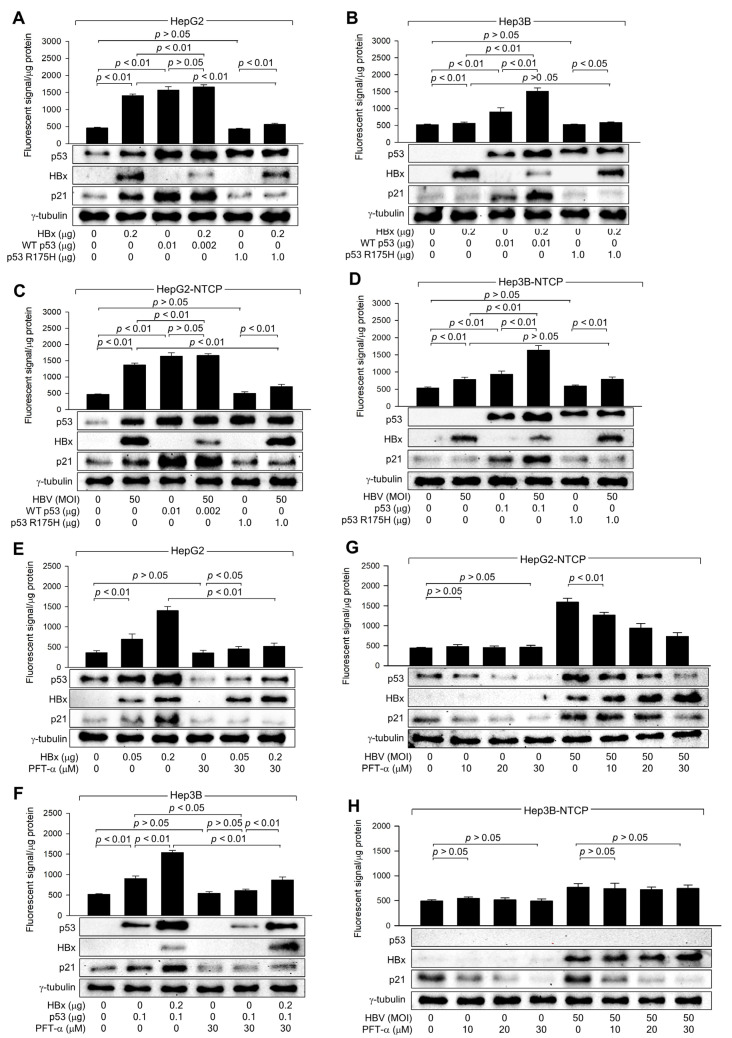
HBx requires p53 transcriptional activity to upregulate ROS levels. (**A**,**B**) HepG2 and Hep3B cells were transfected with the indicated amounts of HBx expression plasmid along with p53 WT and p53 R175H expression plasmid for 48 h, followed by western blotting and measurement of intracellular ROS levels (*n* = 4). (**C**,**D**) HepG2-NTCP and Hep3B-NTCP cells were transfected with p53 WT and p53 R175H expression plasmid for 24 h and then infected with HBV for 3 days, followed by western blotting and measurement of intracellular ROS levels (*n* = 4). (**E**,**F**) HepG2 and Hep3B cells were transfected with the indicated amount of an expression plasmid encoding HBx or p53 for 48 h in the presence or absence of PFT-α, followed by western blotting and measurement of intracellular ROS levels (*n* = 4). (**G**,**H**) HepG2-NTCP and Hep3B-NTCP cells were infected with HBV for 3 days and treated with PFT-α at the indicated concentration for 24 h before harvesting, followed by western blotting and ROS detection assay (*n* = 4). Original western blots are available in the Supplementary Materials.

HBx expression and HBV infection upregulated ROS levels when HBx increased both protein levels and transcriptional activity of p53 in human hepatoma cells (Figure 3), as demonstrated in previous reports [26,32]. To determine whether p53 transcriptional activity is essential for HBx-induced ROS generation, the potential of WT p53 and p53 R175H to upregulate ROS levels in the presence of HBx was compared (Figure 4A,B). Ectopic expression of WT p53 upregulated p21 and ROS levels in both HepG2 and Hep3B cells, regardless of the presence of HBx. HBx also increased both endogenous and exogenous WT p53 in HepG2 and Hep3B cells, leading to an upregulation of p21 and ROS levels (Figure 4A,B). Under all conditions, p53 and p21 levels were consistently proportional to ROS levels. Notably, when WT p53 levels were equalized by transfecting a WT p53 expression plasmid, similar ROS levels were observed in HepG2 cells, with or without HBx (Figure 4A, lanes 3 and 4). The p53 R175H mutation, located within the DNA-binding domain, impairs the ability of p53 to bind DNA, resulting in a loss of its tumor suppressor function, which leads to uncontrolled cell growth and cancer development [38]. Ectopic expression of the p53 R175H mutant failed to upregulate p21 and ROS levels in both HepG2 and Hep3B cells (Figure 4A,B, lane 5). In addition, ectopic expression of p53 R175H significantly reduced the impact of HBx on ROS and p21 levels in HepG2 but not in Hep3B cells (Figure 4A,B, lane 6), presumably due to the role of p53 R175H as a dominant negative mutant [38]. Nonetheless, HBx was still able to upregulate ROS levels in the presence of p53 R175H (Figure 4A,B, lanes 5 and 6), despite its failure to increase p53 and p21 levels. This is likely because HBx induces ROS generation in a p53-independent mechanism involving mitochondrial dysfunction [12,13,39]. The effects of WT p53 and p53 R175H on p53, p21, and ROS levels in HepG2 and Hep3B cells, with or without HBx expression, were exactly reproduced using an in vitro HBV infection system (Figure 4C,D). Therefore, we conclude that p53 transcriptional activity is required to amplify ROS levels induced by HBx during HBV infection in human hepatoma cells.

The necessity of p53 transcriptional activity in the HBx-induced upregulation of ROS levels was further assessed using PFT-α, a specific inhibitor of p53 transcription activity [40]. Treatment with PFT-α downregulated both endogenous and exogenous p53 levels, resulting in a decrease in p21 levels, in HepG2 and Hep3B cells in the presence and absence of HBx (Figure 4E,F). This effect is presumably because p53 transcriptional activity is essential for p53 amplification via a positive feedback loop [41]. Interestingly, PFT-α also downregulated p21 levels in Hep3B cells in a p53-independent manner (Figure 4H), although the underlying mechanism remains unclear. Treatment with PFT-α also downregulated ROS levels as it reduced p53 levels in the presence of HBx (Figure 4E,F); however, this effect was less pronounced in the absence of HBx, possibly due to the detection limit of ROS levels, as demonstrated in p53 knockdown experiments (Figure 3E).

Consequently, the effect of HBx on ROS levels was significantly lower in PFT-α-treated HepG2 and Hep3B cells (Figure 4E,F). Intriguingly, HBx could still upregulate p53 and ROS levels in the presence of PFT-α (Figure 4E,F), consistent with observations made using the p53 R175H mutant (Figure 4A–D). This suggests that HBx can induce ROS generation through mitochondrial dysfunction and upregulate p53 levels via the activation of the ATM-Chk2 pathway without affecting p21 levels in the presence of PFT-α. The effects of PFT-α on p53, p21, and ROS levels in cells with or without HBx expression were exactly reproduced in HBV infection experiments (Figure 4G,H). Based on these observations, we conclude that HBx requires the transcriptional activity of p53 to amplify ROS to high levels in a p53-dependent mechanism.

### 3.5. HBx Downregulates Catalase Levels but Upregulates Mn-SOD Levels via Activation of p53

**Figure 5:**
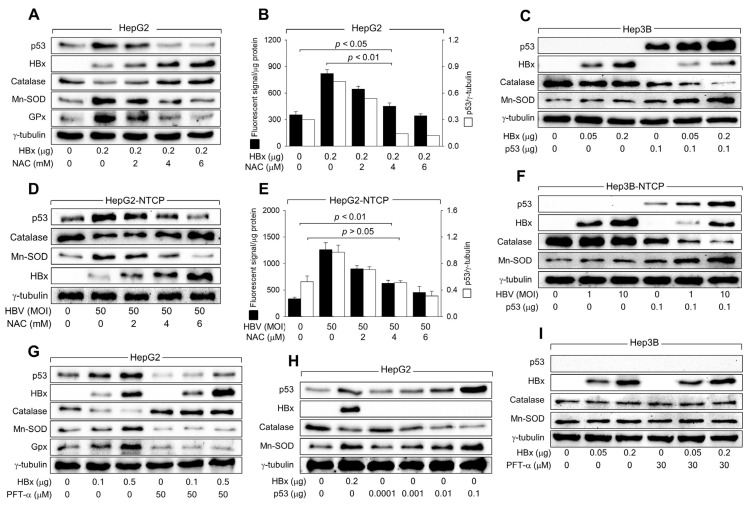
HBx downregulates catalase levels but upregulates Mn-SOD levels via activation of p53. (**A**,**C**) HepG2 and Hep3B cells were transfected with an HBx expression plasmid for 48 h and treated with the indicated concentration of NAC for 24 h before harvesting, followed by western blotting. (**D**,**F**) HepG2-NTCP and Hep3B-NTCP cells were infected with HBV for 24 h and incubated for an additional 3 days, and treated with the indicated concentration of NAC for 24 h before harvesting, followed by western blotting. (**B**,**E**) Levels of ROS in cells prepared in (**A**,**D**) were determined (*n* = 4). The protein bands of p53 and γ-tubulin in (**A**,**D**) were quantified using Image J image analysis software version 1.8.0 (NIH) to show the level of p53 relative to the loading control (γ-tubulin) (*n* = 1 for (**B**) and 3 for (**E**)). (**G**,**I**) HepG2 and Hep3B cells were transfected with HBx expression plasmid for 48 h and treated with PFT-α for 24 h before harvesting, followed by western blotting. (**H**) HepG2 cells were transfected with HBx expression plasmid and p53 expression plasmid for 48 h, followed by western blotting. Original western blots are available in the Supplementary Materials.

We investigated whether HBx affects the levels of major intracellular antioxidant enzymes, including Mn-SOD, catalase, and GPx, to upregulate ROS levels in human hepatoma cells in a p53-dependent manner. According to data obtained using the ectopic HBx expression system, HBx downregulated catalase levels but upregulated Mn-SOD and GPx levels under conditions that also elevated p53 and ROS levels in HepG2 cells (Figure 5A,B). In contrast, ectopic HBx expression minimally affected catalase and Mn-SOD levels in Hep3B cells (Figure 5C). Ectopic expression of p53 enabled HBx to alter catalase and Mn-SOD levels in Hep3B cells (Figure 5C). Consistently, HBV infection upregulated p53, Mn-SOD, and ROS levels, while downregulating catalase levels in both HepG2-NTCP and Hep3B-NTCP cells in a p53-dependent manner. (Figure 5D–F). These results suggest that HBx upregulates Mn-SOD levels while downregulating catalase levels to elevate ROS levels during HBV infection in a p53-dependent mechanism.

The necessity of p53 transcriptional activity in regulating catalase, Mn-SOD, and GPx levels by HBx was further confirmed by inhibiting p53 activity using PFT-α. Treatment with PFT-α almost abolished the ability of HBx to downregulate catalase levels and upregulate Mn-SOD and GPx levels in HepG2 cells (Figure 5G), significantly impairing the capacity of HBx to upregulate ROS levels (Figure 4E). Moreover, transient p53 expression without HBx downregulated catalase levels but upregulated Mn-SOD levels in HepG2 cells in a dose-dependent manner (Figure 5H). Interestingly, similar levels of catalase and Mn-SOD were detected when ectopic expression of either HBx or p53 produced comparable levels of p53 in HepG2 cells (Figure 5H, lanes 2 and 5). The effects of HBx on catalase and Mn-SOD levels, with and without PFT-α, were not observed in Hep3B cells (Figure 5I). Therefore, we conclude that HBx upregulates ROS levels during HBV infection by increasing Mn-SOD levels, which catalyzes the dismutation of O_2_^·–^ to H_2_O_2_, while downregulating catalase levels, which decompose H_2_O_2_ into H_2_O and O_2_, in a p53-dependent mechanism.

### 3.6. HBx Initiates ROS Generation via a p53-Independent Mechanism

Initial experiments showed that both HBx expression and HBV infection elevate ROS levels even in the absence of p53, as demonstrated in Hep3B and Hep3B-NTCP cells, which lack functional p53 (Figure 1 and Figure 2). Additionally, in HepG2 cells where p53 was knocked down below basal levels, ROS generation still occurred following HBV infection or HBx expression (Figure 3A,E). These findings suggest that HBx can trigger ROS production through a p53-independent mechanism, potentially involving mitochondrial dysfunction—a known effect of HBx, as supported by earlier studies [12,13,39]. To further understand the roles of HBx in the initial ROS induction via p53-independent mechanisms and subsequent amplification via activation of p53, we treated HBx-expressing HepG2 cells and HBV-infected HepG2-NTCP cells with NAC, an antioxidant that neutralizes ROS. NAC treatment almost abolished the HBx and HBV-mediated upregulation of p53 levels, removing their effects on catalase, Mn-SOD, and GPx levels in HepG2 cells (Figure 5A,D), indicating that p53 activation plays a crucial role in the subsequent downregulation of catalase and upregulation of Mn-SOD and GPx. However, NAC did not eliminate the ROS generated by HBx and HBV, even though it did reduce ROS levels dose-dependently (Figure 5B,E). The persistent ROS, despite NAC treatment, suggests that the initial ROS generation by HBx, which occurs before the full activation of p53, involves mechanisms that do not depend on p53.

### 3.7. The Ser-101 Residue of HBx Is Critical for ROS Amplification via Activation of p53

**Figure 6:**
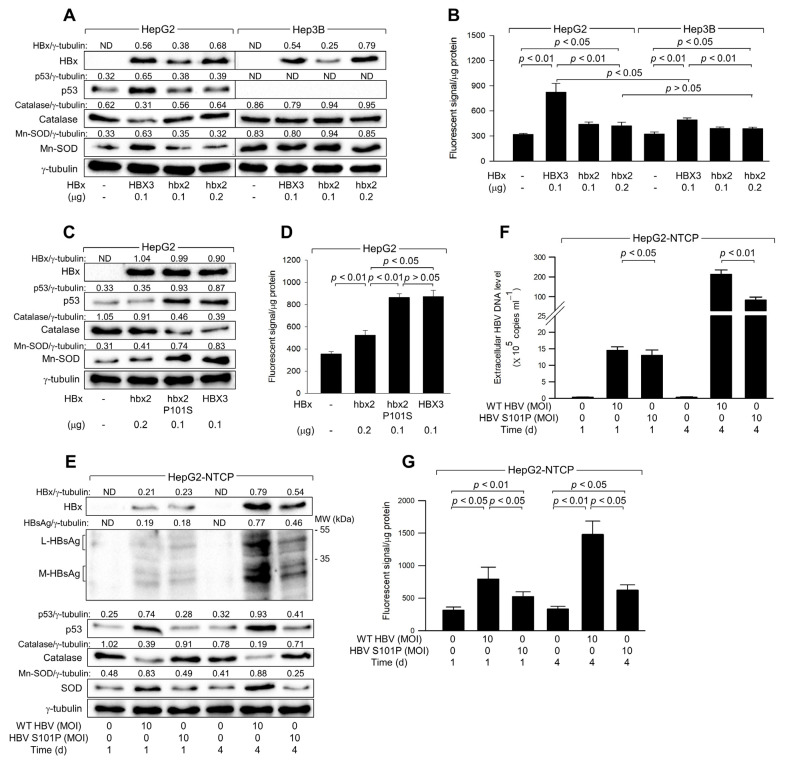
The Ser-101 residue of HBx is critical for ROS amplification via activation of p53. (**A**–**D**) HepG2 and Hep3B cells were transfected with an empty vector or an expression plasmid encoding HBx variants, HBX3, hbx2, or hbx2 P101S, for 48 h. (**A**,**C**) Levels of the indicated proteins in cell lysates were determined by western blotting. (**B**,**D**) Levels of ROS were determined with the indicated conditions (*n* = 4). (**E**,**G**) HepG2-NTCP cells were infected with either HBV WT or HBV S101P for 24 h and incubated for an additional 3 days. (**E**) Levels of the intracellular proteins in cell lysates were determined by western blotting. (**F**) Levels of extracellular HBV DNA in the culture media were determined (*n* = 4). (**G**) Levels of ROS were determined (*n* = 4) at the indicated conditions. Original western blots are available in the Supplementary Materials.

Natural variants of HBx derived from chronic hepatitis and HCC patients contain either Serine (Ser) or Proline (Pro) at the 101st amino acid residue, which is critical for the ability of HBx to upregulate p53 levels [28]. Consistent with previous studies [28], HBx variants such as HBX3, which contains Ser-101 and was widely used in the present study, significantly upregulated p53 levels in HepG2 cells (Figure 6A, lane 2). In contrast, HBx variants like hbx2, containing Pro-101, minimally affected p53 levels despite having similar expression levels of HBx (Figure 6A, lane 4). Furthermore, HBX3, but not hbx2, downregulated catalase levels and upregulated Mn-SOD levels in HepG2 cells (Figure 6A). Consequently, HBX3 demonstrated a much higher potential to upregulate ROS levels compared to hbx2, although both variants could still elevate ROS levels in HepG2 cells (Figure 6B). The ability of HBX3 to upregulate ROS levels was significantly lower in Hep3B cells compared to HepG2 cells, whereas hbx2 showed no clear difference in ROS upregulation between the two cell lines due to its failure to upregulate p53 levels in HepG2 cells (Figure 6A,B). Although both HBX3 and hbx2 significantly upregulated ROS levels in Hep3B cells, HBX3 had a higher potential to induce p53-independent ROS generation than hbx2 (Figure 6B), suggesting that the Ser-101 residue in HBx is essential for upregulating ROS levels by enhancing p53 levels and inducing mitochondrial dysfunction in human hepatoma cells.

To confirm the critical role of the Ser-101 residue in the ability of HBx to upregulate p53 and ROS levels, we utilized a mutant variant, hbx2 P101S, where the Ser residue is artificially substituted for Pro at position 101 in the hbx2 variant [28]. Unlike the original hbx2 variant, hbx2 P101S was able to upregulate p53 to levels comparable to those observed with the HBX3 variant, leading to a subsequent downregulation of catalase and an upregulation of Mn-SOD in HepG2 cells (Figure 6C, lane 3). Consistent with these observations, both HBX3 and hbx2 P101S similarly upregulated ROS levels, which were distinctly higher than those produced by hbx2 (Figure 6D). These results underscore the crucial role of the Ser-101 residue in HBx for the upregulation of p53 and ROS levels in human hepatoma cells.

Consistent with the observations from the HBx overexpression system (Figure 6A–D), infection with WT HBV, which encodes HBx with the Ser-101 residue, resulted in a time-dependent upregulation of p53 levels in HepG2-NTCP cells (Figure 6E). This upregulation of p53 was accompanied by a decrease in catalase levels and an increase in Mn-SOD levels, leading to elevated ROS levels during infection in HepG2-NTCP cells (Figure 6E,G). In contrast, infection with HBV S101P, where Ser residue is replaced with Pro-101 in HBx [42], showed minimal effects on p53, catalase, and Mn-SOD levels in HepG2-NTCP cells (Figure 6E). Despite similar levels of HBx expression in both WT HBV and HBV S101P-infected cells after 1 day of infection, the effects of WT HBV on p53, Mn-SOD, catalase, and ROS levels were significantly higher (Figure 6E,G), highlighting the crucial role of the Ser-101 residue in HBx for p53 activation and the consequent upregulation of ROS levels. However, HBV S101P could increase ROS levels during infection in HepG2-NTCP cells (Figure 6E), which provides evidence for the HBx-induced ROS generation through p53-independent mechanisms [12,13,39]. Additionally, consistent with an earlier report [42], WT HBV replicated more effectively than HBV S101P in HepG2-NTCP cells, as evidenced by higher levels of intracellular HBV proteins, including HBx and HBsAg, and more extracellular HBV particles (Figure 6E,F). Therefore, the Ser-101 in HBx appears to be essential for the upregulation of p53 and ROS levels and stimulation of HBV replication.

## 4. Discussion

HBV infection typically induces oxidative stress, characterized by elevated levels of ROS such as H_2_O_2_ in the liver and blood of patients [4,5,6]. HBV itself contributes to ROS accumulation in hepatocytes [8,9,10]. This study demonstrated that HBV infection increases ROS levels in human hepatoma cells (Figure 1A,B). Previous reports have demonstrated the role of HBx in ROS generation during HBV infection [8,9,10]. Consistently, while HBx-null HBV could induce ROS generation—presumably due to the possible roles of other viral proteins such as HBsAg and HBcAg [43]— the effect is significantly weaker compared to HBx-expressing HBV (Figure 2E), underscoring the predominant role of HBx in ROS generation during infection. Moreover, ectopic expression of HBx alone was sufficient to increase ROS levels in human hepatoma cells (Figure 2G).

This study reveals that HBx induces ROS through a two-step process: first, by initiating ROS generation via a p53-independent mechanism, and second, by amplifying ROS through a p53-dependent pathway. Several lines of evidence support that HBx can trigger ROS production through a p53-independent mechanism, potentially involving mitochondrial dysfunction—a known effect of HBx, as supported by earlier studies [11,12,13,14,15]. First, both HBx expression and HBV infection elevate ROS levels even without p53, as demonstrated in Hep3B and Hep3B-NTCP cells (Figure 1 and Figure 2). Second, in HepG2 cells where p53 was knocked down below basal levels, ROS generation occurred following HBV infection or HBx expression (Figure 3A,E). Third, HBx expression and HBV infection could induce ROS generation under conditions that either the p53 R175 mutant or PFT-α completely inhibited p53 transcriptional activity (Figure 4). Fourth, ROS removal using NAC did not eliminate the potential of HBx to generate ROS, even though it completely abolished its ability to activate p53 (Figure 5). Fifth, HBx variants containing Pro-101 could upregulate ROS levels, even though they failed to activate p53 in both HBx expression and HBV infection systems (Figure 6B). Therefore, the initial step for ROS generation by HBx occurs before the full activation of p53 during HBV infection.

The second phase by which HBx increases ROS levels involves the amplification of ROS in a p53-dependent mechanism. Previous studies have revealed that ROS acts as an upstream signal that triggers p53 activation and a downstream factor amplified by p53 via a positive feedback loop [21]. Previous reports have demonstrated that HBx-induced ROS causes DNA damage, such as double-strand DNA breaks, resulting in activation of the ATM-Chk2 pathway and subsequent stabilization of p53 via phosphorylation at the Ser-15 and Ser-20 residues [44,45]. The present study also showed that either ectopic HBx expression or HBV infection increases ROS and p53 levels in hepatoma cells (Figure 1 and Figure 2). In addition, the present study provides several lines of evidence supporting that HBx increases ROS levels via activation of p53 during HBV infection. First, both HBx expression and HBV infection significantly induced ROS generation in HepG2 and HepG2-NTCP cells, respectively, whereas this effect was much lower in Hep3B and Hep3B-NTCP cells, where p53 was absent (Figure 1G and Figure 2G). Second, intracellular ROS levels, which were increased by both HBx expression and HBV infection, gradually decreased as p53 was knocked down in HepG2 and HepG2-NTCP cells (Figure 3A,E). These experiments may also exclude the possible involvement of other differences between HepG2 and Hep3B cells, including ethnic origins, distinct chromosome aberrations, HBV DNA integration, and tumorigenicity [31,46], in ROS generation during HBV infection. Third, ROS levels, which were not responsive to HBV infection in Hep3B and Hep3B-NTCP cells, were significantly increased by p53 expression and were further elevated upon HBx expression and HBV infection (Figure 3B,F). Fourth, ectopic expression of p53 without HBx involvement was sufficient to induce ROS generation in HepG2 and Hep3B cells (Figure 3C,D). Fifth, NAC treatment abolished the HBx and HBV-mediated upregulation of p53 levels, indicating that ROS generation was necessary for p53 activation (Figure 5A,D). Sixth, both p53 and ROS levels were upregulated by HBx variants containing Ser-101, but not by those with Pro-101, in both HBx expression and HBV infection systems (Figure 6), demonstrating the direct correlation between the potential of HBx to activate p53 and its ability to upregulate ROS levels.

The p53 transcriptional activity appears to be essential for the ROS amplification step. Unlike WT p53, p53 R175H failed to increase ROS levels in the presence and absence of HBx in both HepG2 and Hep3B cells (Figure 4A,B). In addition, treatment with PFT-α severely impaired the ability of HBx to induce ROS generation in HepG2 and Hep3B cells (Figure 4E,F). Moreover, the intracellular ROS levels were directly proportional to levels of p21, a representative p53 target (Figure 3 and Figure 5). Therefore, the HBx-mediated upregulation of ROS levels may involve transcriptional regulation of p53 target genes, which are directly or indirectly associated with ROS generation. The present study provides several lines of evidence supporting that HBx-activated p53 downregulates catalase and upregulates Mn-SOD levels, resulting in upregulation of ROS levels during infection in human hepatoma cells. First, HBx expression and HBV infection downregulated catalase levels and upregulated GPx levels in HepG2 and HepG2-NTCP cells, while these effects were negligible in Hep3B and Hep3B-NTCP cells (Figure 5A–F). The ability of HBx to alter catalase and Mn-SOD levels and upregulate ROS levels simultaneously disappeared when the potential of HBx to activate p53 was abolished by treatment with either NAC or PFT-α (Figure 5A,B,D,E,G,I). Third, HBx variants with Ser-101 residue could upregulate p53, Mn-SOD, and ROS levels and downregulate catalase levels, whereas these effects were much lower or negligible with HBx variants containing Pro-101 residue (Figure 6). Fourth, ectopic p53 expression in the absence of HBx was sufficient to upregulate ROS levels and alter catalase and Mn-SOD levels in HepG2 cells (Figure 5H).

The detailed mechanism by which p53 regulates the expression of catalase and Mn-SOD during HBV infection is still elusive. It might be easy to explain the HBx-mediated upregulation of Mn-SOD and GPx levels because genes encoding these proteins contain p53-binding sites in the promoters and are thus known to be responsive to p53 for their basal and induced expression [23,24]. However, it is unclear how HBx and p53 downregulate catalase expression, although its promoter also contains a putative p53 binding site [25]. In addition, it is unclear how the upregulation of GPx, which plays an important role in eliminating ROS [47], contributes to oxidative stress induced by HBx. It may simply represent an outcome of p53 activation and does not contribute to the accumulation of ROS during HBV infection. Further studies are required to elucidate the precise mechanism by which the HBx and p53 regulate levels of antioxidant enzymes and their relative contributions to ROS accumulation during HBV infection. In conclusion, HBx initiates ROS production via a p53-independent pathway, likely through mechanisms such as mitochondrial dysfunction. Following this initial induction, ROS levels are further amplified by p53-dependent mechanisms, which include the transcriptional regulation of genes involved in oxidative stress management. This dual mechanism underscores the complex role of HBx in modulating cellular oxidative stress during HBV infection, which may contribute to the pathogenesis of liver diseases such as hepatocellular carcinoma.

## Data Availability

The data presented in this study are available from the corresponding author upon reasonable request.

## Supplementary Materials

The following supporting information can be downloaded at: https://www.mdpi.com/article/10.3390/biom14101201/s1, Original western blots are available in the Supplementary Materials.
