# Supplementary material for: Hepatitis B Virus X Protein Induces Reactive Oxygen Species Generation via Activation of p53 in Human Hepatoma Cells
Source: Biomolecules. 2024 Sep 24;14(10):1201. doi: 10.3390/biom14101201 (PMC11505488; doi:10.3390/biom14101201)

Figure 1A

	HepG2-NTCP				Hep3B-NTCP			
lane	1	2	3	4	5	6	7	8
HBV (MOI)	0	50	50	50	0	50	50	50
Time (d)	0	1	4	7	0	1	4	7

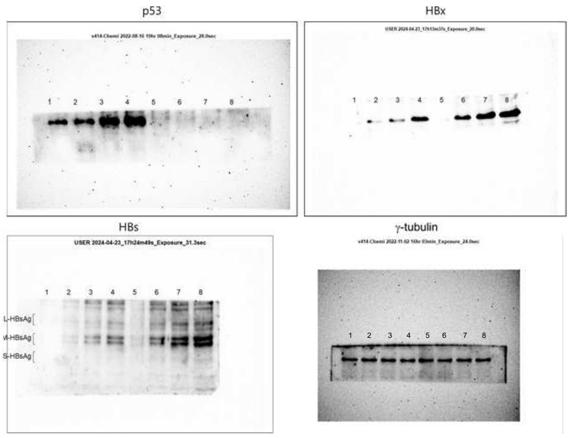

Figure 1B

	HepG2-NTCP			Hep3B-NTCP		
lane	1	2	3	4	5	6
HBV (MOI)	0	10	50	0	10	50
Time (d)	0	4	4	0	4	4

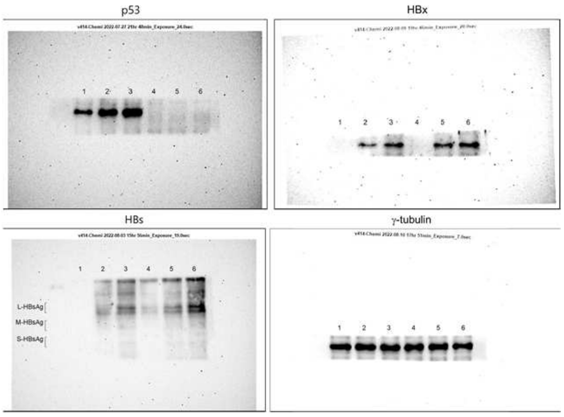

Figure 1J

	HepG2			
lane	1	2	3	4
HBV (MOI)	0	50	0	50
Time (d)	4	4	4	4
MyrB (μM)	0	0	1	1

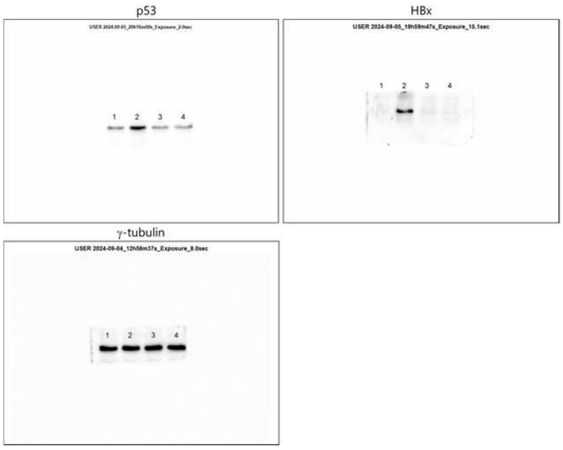

Figure 2A

	HepG2			Hep3B		
lane	1	2	3	4	5	6
1.2-mer WT (µg)	0	0.1	0.5	0	0.1	0.5

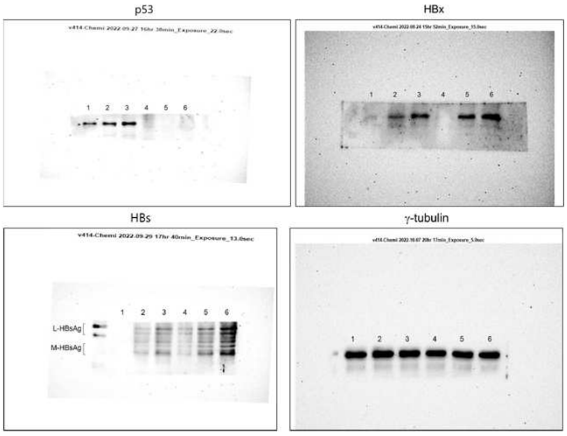

Figure 2C

	HepG2			Hep3B		
Lane	1	2	3	4	5	6
1.2-mer WT (µg)	0	0.5	0	0	0.5	0
1.2-mer HBx-null (µg)	0	0	0.5	0	0	0.5
HBx (µg)	0	0	0	0	0	0

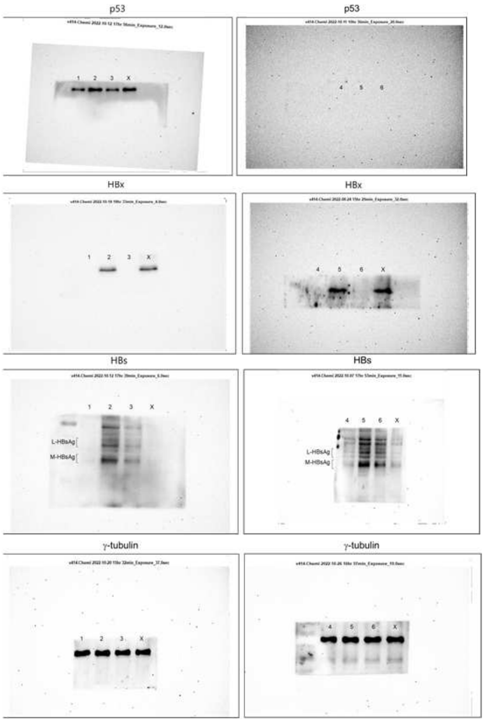

Figure 2F

	HepG2			Hep3B		
Lane	1	2	3	4	5	6
HBx(µg)	0	0.05	0.2	0	0.05	0.2

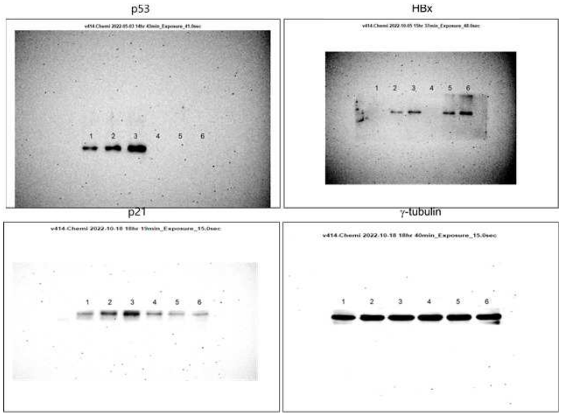

Figure 3A

	HepG2					
lane	1	2	3	4	5	6
HBx (µg)	0	0	0	0.2	0.2	0.2
SC shRNA (µg)	1.0	0.9	0	1.0	0.9	0
p53 shRNA (µg)	0	0.1	1.0	0	0.1	1.0

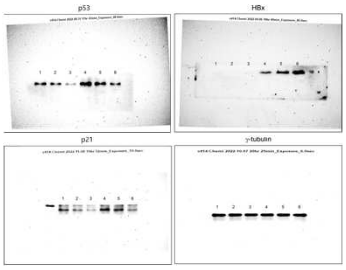

Figure 3B

	Hep3B					
lane	1	2	3	4	5	6
HBx (µg)	0	0.05	0.2	0	0.05	0.2
p53 (µg)	0	0	0	0.1	0.1	0.1

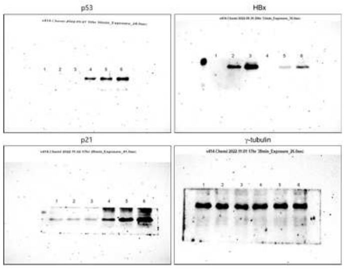

Figure 3C

	HepG2					
lane	1	2	3	4	5	6
HBx (µg)	0	0.2	0	0	0	0
p53 (ng)	0	0	1	5	25	125

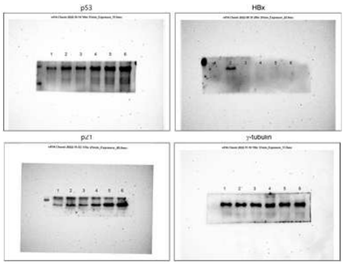

Figure 3D

	Hep3B				
lane	1	2	3	4	5
HBx (µg)	0	0.2	0	0	0
p53 (µg)	0	0	0.05	0.1	0.2

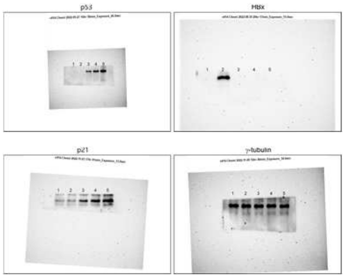

Figure 3E

	HepG2-NTCP					
lane	1	2	3	4	5	6
HBV (MOI)	0	0	0	50	50	50
SC shRNA	1.0	0.9	0	1.0	0.9	0
p53 shRNA (µg)	0	0.1	1.0	0	0.1	1.0

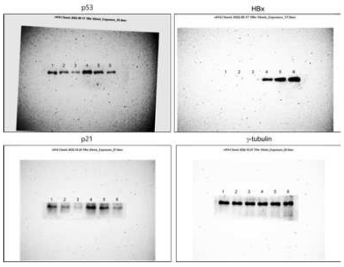

Figure 3F

	Hep3B-NTCP					
lane	1	2	3	4	5	6
p53 (µg)	0	0.05	0.1	0.2	0	0.05
HBV (MOI)	0	0	0	0	50	50

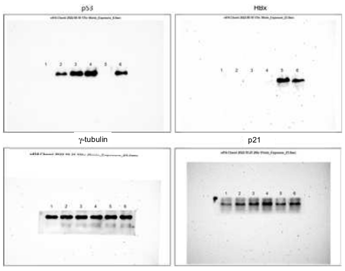

Figure 4A

HepG2						
lane	1	2	3	4	5	6
HBx (μg)	0	0.2	0	0.2	0	0.2
WT p53 (μg)	0	0	0.01	0.002	0	0
p53 R175H (μg)	0	0	0	0	1.0	1.0

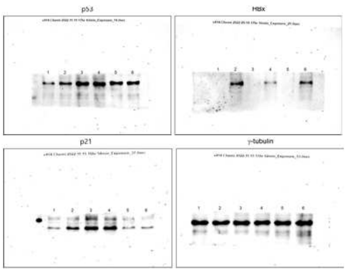

Figure 4B

Hep3B						
lane	1	2	3	4	5	6
HBx (μg)	0	0.2	0	0.2	0	0.2
WT p53 (μg)	0	0	0.01	0.01	0	0
p53 R175H (μg)	0	0	0	0	1.0	1.0

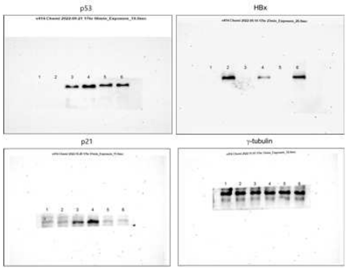

Figure 4C

HepG2-NTCP						
lane	1	2	3	4	5	6
HBV (MOI)	0	50	0	50	0	50
WT p53 (μg)	0	0	0.01	0.002	0	0
p53 R175H (μg)	0	0	0	0	1.0	1.0

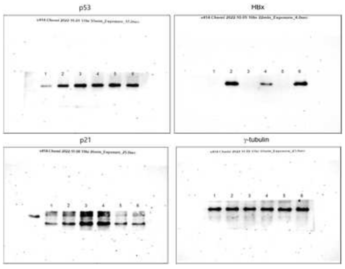

Figure 4D

Hep3B-NTCP						
lane	1	2	3	4	5	6
HBV (MOI)	0	50	0	50	0	50
p53 (μg)	0	0	0.1	0.1	0	0
p53 R175H (μg)	0	0	0	0	1.0	1.0

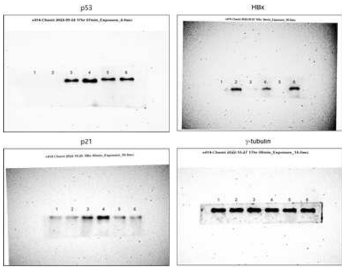

Figure 4E

HepG2						
lane	1	2	3	4	5	6
HBx (μg)	0	0.05	0.2	0	0.05	0.2
PFT-α (μM)	0	0	0	30	30	30

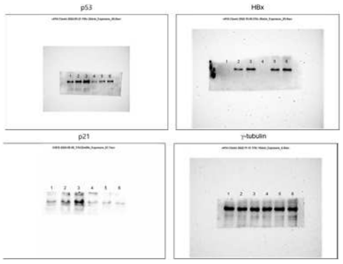

Figure 4G

HepG2-NTCP								
lane	1	2	3	4	5	6	7	8
HBV (MOI)	0	0	0	0	50	50	50	50
PFT-α (μM)	0	10	20	30	0	10	20	30

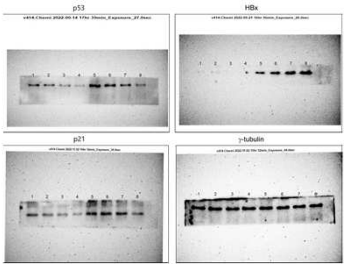

Figure 4F

Hep3B						
lane	1	2	3	4	5	6
HBx (μg)	0	0	0.2	0	0	0.2
p53 (μg)	0	0.1	0.1	0	0.1	0.1
PFT-α (μM)	0	0	0	30	30	30

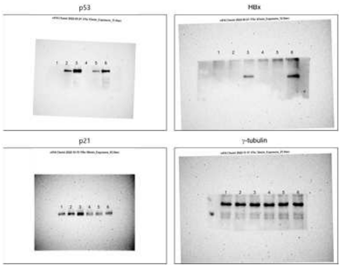

Figure 4H

Hep3B-NTCP								
lane	1	2	3	4	5	6	7	8
HBV (MOI)	0	0	0	0	50	50	50	50
PFT-α (μM)	0	10	20	30	0	10	20	30

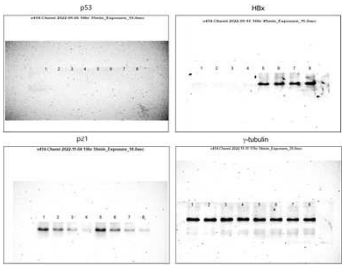

Figure 5A

HepG2					
lane	1	2	3	4	5
HBx (μg)	0	0.2	0.2	0.2	0.2
NAC (mM)	0	0	2	4	6

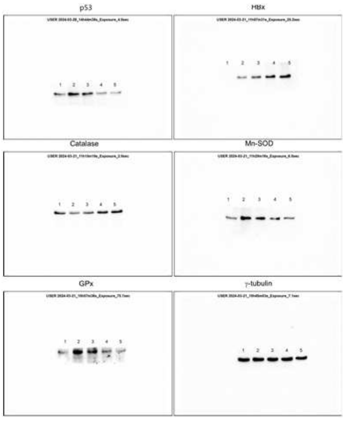

Figure 5C

Hep3B						
lane	1	2	3	4	5	6
HBx (μg)	0	0.05	0.2	0	0.05	0.2
p53 (μg)	0	0	0	0.1	0.1	0.1

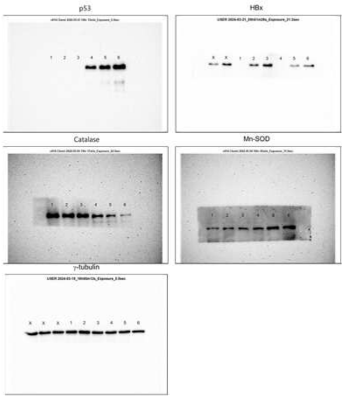

Figure 5D

HepG2-NTCP					
lane	1	2	3	4	5
HBV (MOI)	0	50	50	50	50
NAC (mM)	0	0	2	4	6

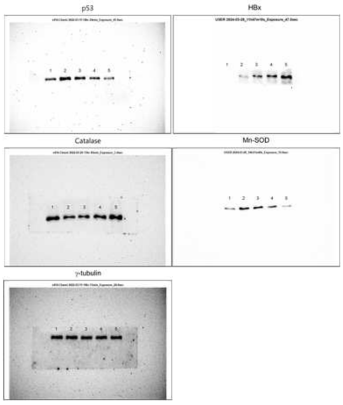

Figure 5F

Hep3B-NTCP						
lane	1	2	3	4	5	6
HBV (MOI)	0	1	10	0	1	10
p53 (μg)	0	0	0	0.1	0.1	0.1

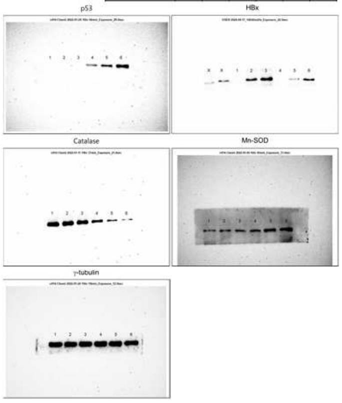

Figure 5G

HepG2						
lane	1	2	3	4	5	6
HBx (μg)	0	0.05	0.2	0	0.05	0.2
PFT-α (μM)	0	0	0	30	30	30

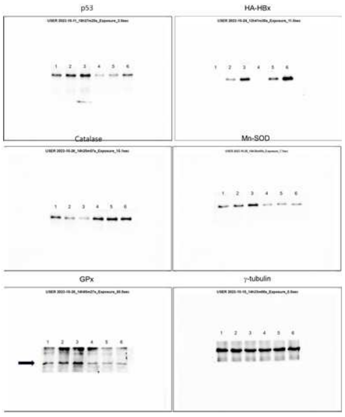

Figure 5H

HepG2						
lane	1	2	3	4	5	6
HBx (μg)	0	0.2	0	0	0	0
p53 (μg)	0	0	0.0001	0.001	0.01	0.1

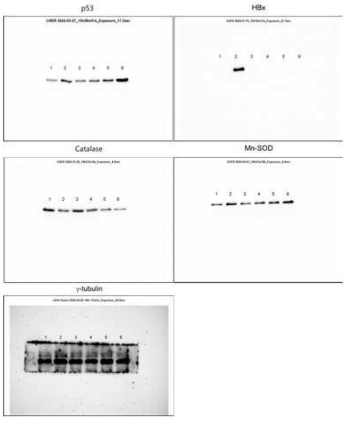

Figure 5I

Hep3B						
lane	1	2	3	4	5	6
HBx (μg)	0	0.05	0.2	0	0.05	0.2
PFT-α (μM)	0	0	0	30	30	30

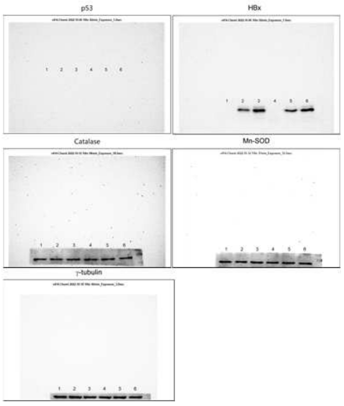

Figure 6A

	HepG2				Hep3B			
lane	1	2	3	4	5	6	7	8
HBx	-	HBX3	hbx2	hbx2	-	HBX3	hbx2	hbx2
(μg)	-	0.1	0.1	0.2	-	0.1	0.1	0.2

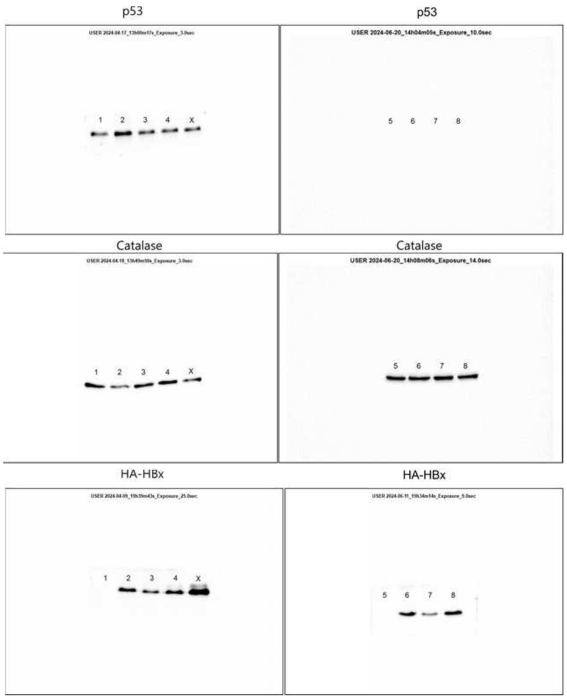

Figure 6A

	HepG2				Hep3B			
lane	1	2	3	4	5	6	7	8
HBx	-	HBX3	hbx2	hbx2	-	HBX3	hbx2	hbx2
(μg)	-	0.1	0.1	0.2	-	0.1	0.1	0.2

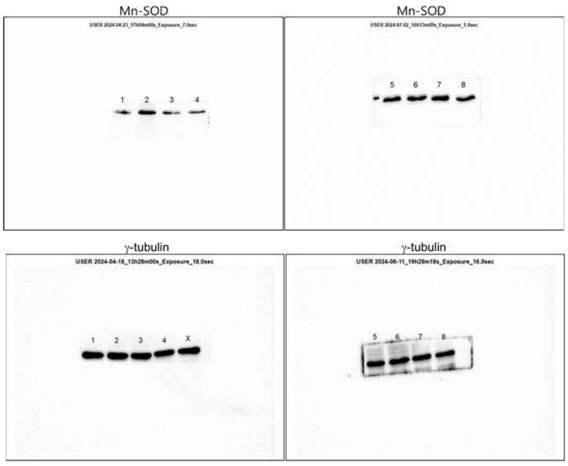

Figure 6C

	HepG2			
lane	1	2	3	4
HBx	-	hbx2	hbx2 P101S	HBX3
(μg)	-	0.2	0.1	0.1

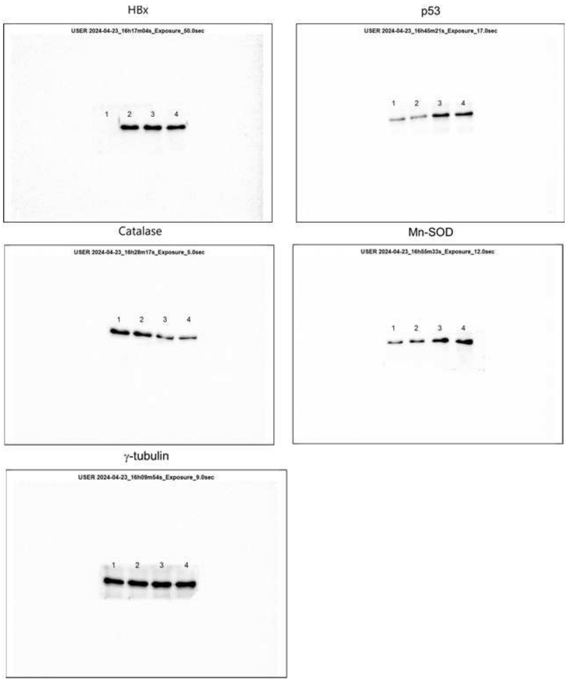

Figure 6E

	HepG2-NTCP					
lane	1	2	3	4	5	6
WT HBV (MOI)	0	10	0	0	10	0
HBV S101P (MOI)	0	0	10	0	0	10
Time (day)	1	1	1	4	4	4

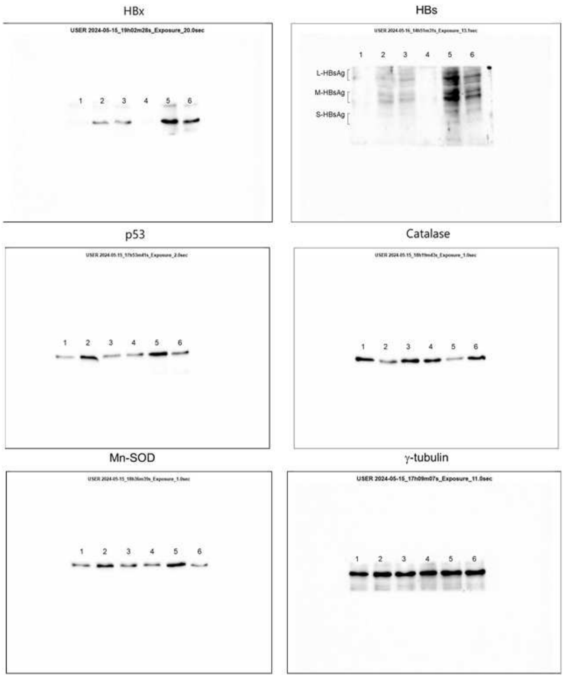

Supplement: Supplementary file 1 [file biomolecules-14-01201-s001.zip › biomolecules-3173344-supplementary.pdf]
